# A singular case of hyposmia and transient audiovestibular post-vaccine disorders: case report and literature review

**DOI:** 10.1515/tnsci-2022-0250

**Published:** 2022-10-07

**Authors:** Francesco Fantin, Andrea Frosolini, Isabella Tundo, Ingrid Inches, Cristoforo Fabbris, Giacomo Spinato, Cosimo de Filippis

**Affiliations:** Department of Neuroscience DNS, University of Padova, Audiology Unit, Treviso Hospital, Treviso, Italy; Department of Medical and Surgical Sciences and Advanced Technologies “G.F. Ingrassia,” ENT Section, A.O.U. Policlinico “G.Rodolico-San Marco,” University of Catania, Catania, Italy; Department of Diagnostic Imaging, Neuroradiology Unit, Treviso Hospital, Treviso, Italy; Department of Neuroscience DNS, University of Padova, Otolaryngology Unit, Treviso Hospital, Treviso, Italy

**Keywords:** tinnitus, smell and taste disorders, vaccine adverse reaction, COVID-19 vaccination, olfactory dysfunction, anosmia, hyposmia, olfactory bulb

## Abstract

**Introduction:**

Rare and mild adverse effects on cranial nerves have been reported after vaccination. Here, we report a singular case of smell and taste disorder associated with tinnitus that occurred after Oxford-AstraZeneca vaccination together with a review of the available literature.

**Case presentation:**

A 76-year-old patient experienced smell disorder, ear fullness and tinnitus 2 days after the first dose of Oxford-AstraZeneca vaccine. The patient then underwent a complete audiological and Ear, Nose and Throat evaluation, nasal endoscopy, Sniffin’Sticks battery, audiometric test battery, and cerebral magnetic resonance imaging (MRI). The exams revealed hyposmia and bilateral reduction of the volume of the olfactory bulbs (OB). At the follow-up, tinnitus was completely resolved while olfactory dysfunction only partially reduced.

**Review of the literature:**

A PubMed search was conducted on olfactory and gustatory dysfunctions after COVID-19 vaccination resulting in four case reports with a total of 10 patients. The main symptoms were hyposmia, parosmia, and dysgeusia developed after 1–9 days from vaccination with complete resolution occurring within 1 month. Notably, none of the considered articles reported reduction of OB volumes at cerebral MRI.

**Discussion:**

So far, no definitive cause–effect relationship has been established between anti-COVID19 vaccination and otolaryngologic adverse reactions. The persistence of hyposmia in our patient could possibly be explained by the reduction in OB volume, even though also the advanced age of the patient needs to be taken into account. This is a first indication of a cause–effect relation between hyposmia and Covid19 vaccination, even though a more robust study is needed to confirm the autoimmunological mechanisms responsible for these rare adverse reactions. However, it is worth highlighting that benefits of the anti-COVID-19 vaccination clearly outweigh the risk of rare adverse events.

## 1 Introduction

Transient olfactory and gustatory dysfunctions have recently been reported to possibly represent a rare side effect of anti-coronavirus disease 2019 (COVID-19) vaccines [1]. Other main side effects on cranial nerves after anti-COVID-19 vaccination are Bell palsy and audiovestibular disorders [2,3]. Even if no clear explanations have been given yet, vascular events or abnormal immune responses have been pointed out, thus justifying even possible rare and unexpected issues [3]. Herein, we reported a singular case of smell and taste disorders associated with tinnitus after Oxford-AstraZeneca vaccination and we also consulted the international literature regarding olfactory and gustatory dysfunctions after vaccination.

## 2 Patients and methods

The patient was evaluated at the Smell and Taste Clinic of the Otolaryngology-Audiology and Phoniatrics Units, Department of Neuroscience Padua, Treviso. The patient underwent complete ear, nose, and throat evaluation including nasal endoscopy and audiometric test battery.

The Sniffin’Sticks battery test was performed to evaluate olfactory function. Results were interpreted according to the appropriate scoring system considering the patient’s age. Scores were considered “normal” (above 30 points), “hyposmia” (between 30 and 15), and “anosmia” (below 15) [1].

The magnetic resonance imaging (MRI) was acquired using a 3.0 T MR scanner (Magnetom Vida, Siemens Healthcare, Erlangen, Germany) with a 64-channel head and neck coil with a standardized protocol for olfactory tract analysis. The protocol included axial T2W TSE covering the whole brain (matrix 512; FOV 230, 4 mm, TR 4090, TE 74), DWI (matrix 200, FOV 230, 4 mm, TR 2009, TE 66), coronal T2W covering the anterior and middle segments of the skull base (matrix 512, FOV 160, 2 mm, TR 7390, TE 80), cor T2 space isovolumetric (matrix 320, FOV 230, 0.7 mm, TR 1400, TE 158), and isotropic T1W MPR covering the whole brain (matrix 288, FOV 260, 0.9 mm, TR 2200, TE 2.53), isotropic FLAIR 3D (matrix 288, FOV 245, 0.9 mm, TR 8500, TE 386), and isotropic T1W MPR after Gadolinium. All the 3D sequences were reconstructed in coronal, axial, and sagittal projection for the radiologic evaluation. Intensity of olfactory bulbs (OB) is defined as normal when bulbs have the same cortical intensity, as typically seen in healthy controls. Abnormal OBs’ intensity is defined when the bulb is more hyperintense than the cortex on T2WI and FLAIR. After gadolinium injection on T1WI, enhancement of the OBs was defined when they become more hyperintense in comparison with their intensity on pre-gadolinium T1WI [4].

Boundaries of OB were determined by using the surrounding cerebrospinal fluid and the anterior cribriform plate as markers. Atrophy reduction of OB was diagnosed according to the following findings: flattering and thinning of the OB with the loss of the normal oval shape and an asymmetric decrease in the size of one OB compared with the contralateral side. OB volume was calculated by measuring the planimetric manual contouring of the OB obtaining the surface in mm²; after that all the surfaces were added and multiplied by the thickness of the slices. Posterior end of the OB and beginning of the olfactory tract have been determined when the measured surface of two successive slices was the same [5].

**Figure 1:**
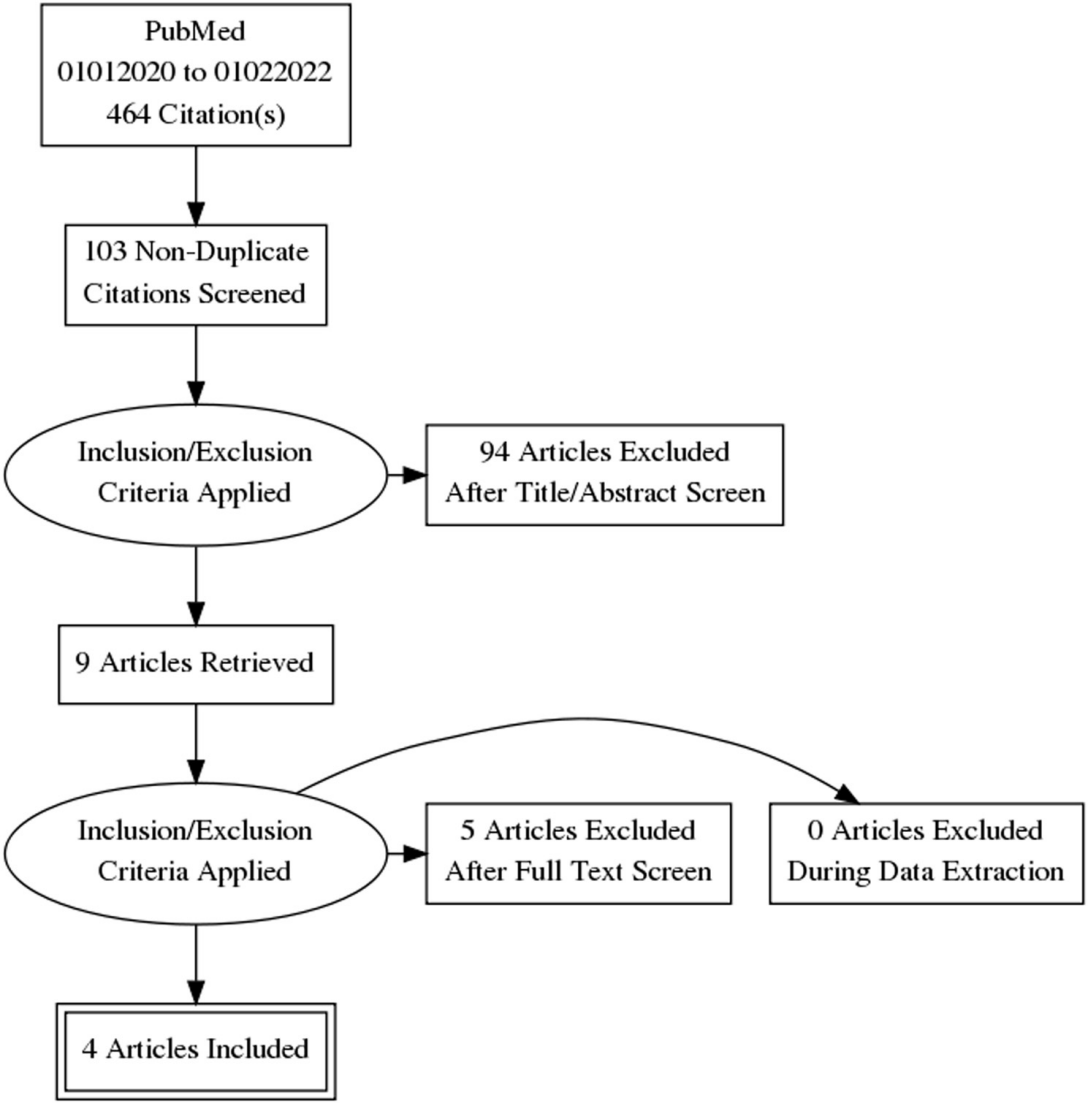
Flow chart of the present literature review on smell and taste alteration after SARS-CoV-2 vaccination.


A structured search through the English literature published on PubMed from 01.01.2020 to 01.02.2022 was conducted. The terms “vaccin hyposmia,” “vaccine smell,” “vaccine olfaction,” “vaccine olfactory,” and “post vaccine anosmia” were used. The reference lists of all the included articles were accurately screened to identify other pertinent studies. The “Related articles” option available on the PubMed homepage was also considered. Only SARS-CoV-2 vaccination-related reports were included. Two of the authors (F.F. and A.F.) independently analyzed the data from the available literature. Any disagreement about inclusion/exclusion of manuscripts was solved by a discussion among the study team members. The PRISMA diagram (Figure 1) resumes the search strategy and the retrieved results.

**Ethical approval:** The research related to human use has been complied with all the relevant national regulations, institutional policies and in accordance the tenets of the Helsinki Declaration, the Italian privacy and sensitive data laws, the in-house regulations of our hospital and has been approved by the authors’ institutional review board or equivalent committee.
**Informed consent:** Informed consent has been obtained from the individual included in this study.

## 3 Results

**Figure 2:**
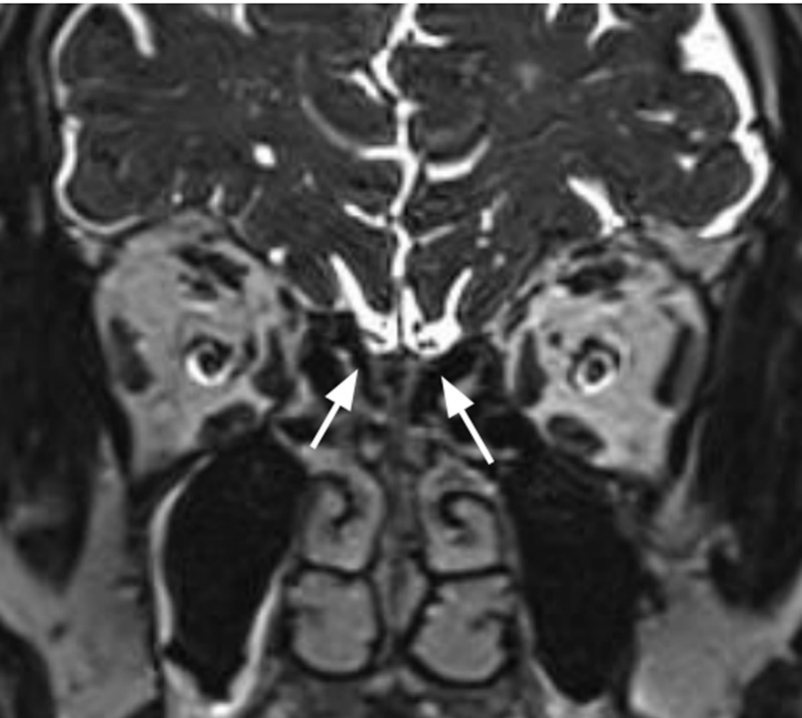
T2WI coronal MRI image of the present case report, with white arrow showing olfactory bulb mild atrophy (right 19.01 mm^3^; left 23.36 mm^3^).

A 76-year-old male underwent the first dose of the anti-COVID-19 vaccination on March 31, 2021, with Oxford-Astrazeneca vaccine. Two days later, he complained of hyposmia, dysgeusia, and parosmia, left aural fullness, and tinnitus. Nasopharyngeal swab tested negative for Severe-Acute-Respiratory-Syndrome-Coronavirus-2 (SARS-Co-V-2). After 2 weeks, he underwent a complete audiological evaluation which showed bilateral presbycusis; otoscopy, auditory brainstem response, and impedance test resulted within normal limits. He was also evaluated for smell and taste disturbances: video-rhinolaryngoscopy did not reveal any alteration; the result of Sniffin’Sticks Test (Burghart GmbH, Wedel, Germany) was consistent with hyposmia (Threshold – T: 1.5/16; Discrimination – D: 11/16; Identification – I: 7/16; Threshold, Discrimination and Identification sum – TDI: 19.5/48). Nasal cortisone spray, multivitamin supplementation, and olfactory rehabilitation exercises (consisting of self-administration of common odorants 10 min a day for 4 months) were prescribed. At 3-month follow-up after symptom onset, the tinnitus had fully recovered, and the olfactory dysfunction had only partially improved, with the persistence of hyposmia (TDI: 21; T:5; D:8; I:8). A brain MRI (Figure 2) revealed leukoaraiosis (grade 1) and mild atrophy of the OB, without any signal alterations (T2W OB volume: right 19.01 mm^3^; left 23.36 mm^3^).

Table 1 summarizes the main findings of the review. Four studies with a total of 10 patients (8 women and 2 men, age 25–57) were retrieved [1,6,7,8]. Six patients experienced hyposmia [1,8] and three had parosmia [1,6,7]; only one patient complained of dysgeusia [1]. Administered vaccines were Oxford-AstraZeneca and Pfizer-BioNTech. Time elapsed from vaccine to symptom onset ranged from 1 to 9 days [1]. Duration of the symptoms ranged from 4 days [1] to a month [8]. In the only patients who performed cerebral MRI, OB hyperintensity and OC edema were reported [8].

**Table 1:** Epidemiological and clinical characteristics of patients with smell and taste disorders after vaccine

Reference	Patient age, gender	COVID-19	Dose, vaccine	DVSO	Symptoms	Fibroscopy	STT	STT results	STT follow-up	RMN
[1]	25, F	No	1st, Astrazeneca	2	Hyposmia	NR	Sniffin’Sticks	27/48	40/48	NR
	27, F	No	1st, Astrazeneca	2	Hyposmia	NR	Sniffin’Sticks	NR	39/48	NR
	50, F	No	1st, Astrazeneca	2	Hyposmia	Normal	Sniffin’Sticks	NR	NR	NR
	30, F	No	2nd, Pfizer	1	Hyposmia	NR	Sniffin’Sticks	NR	NR	NR
	44, M	No	1st, Astrazeneca	2	Dysgeusia	Normal	16-p Sniff; test strip	15/16; 0/4 (salty)	NR	NR
	33, F	Yes	2nd, Pfizer	9	Parosmia	Normal	16-p Sniff	11/16	NR	NR
[6]	42, F	Yes	2nd, Pfizer	3	Hyposmia	Normal	Sniffin’Sticks	22/48	27/48	NR
	39, F	No	2nd, Pfizer	5	Hyposmia	Normal	Sniffin’Sticks	27/48	34/48	NR
[7]	38, M	Yes	2nd, Astrazeneca	7	Parosmia	Normal	16-p Sniff	7/16	NR	NR
[8]	57, F	No	2nd, Pfizer	NR	Parosmia	NR	NR	NR	NR	OB hyperintensity, OT edema
Present case	76, M	No	1st, Astrazeneca	2	Hyposmia, tinnitus	Normal	Sniffin’Sticks	19.5/48	21/48	OB volume reduction, leukoaraiosis

Abbreviations: DVSO, days from vaccine to symptom onset; NR, not reported; OB, olfactory bulb; OT, olfactory tract; STT, smell and taste tests.

## 4 Discussion and conclusion

Otolaryngologic adverse reactions after anti-COVID-19 vaccination are rare and controversial [9], and pathophysiological mechanisms of such symptoms, including smell and taste dysfunction, remain speculative. A hypersensitivity reaction may be involved, causing an abnormal autoimmune response direct to the olfactory neuroepithelium [1]. On the other hand, immunization anxiety-related reactions have been postulated [2]. Lastly, a coincidental event may have occurred, including SARS-CoV-2 infection at the time of vaccination [3].

According to our review, only 10 cases of smell and taste disturbances occurred after Oxford-AstraZeneca and Pfizer-BioNTech vaccinations have been described in the international literature up to date. Notably, most cases were transient and without associated audiovestibular symptoms, and the only OB alteration previously noted was OB hyperintensity in a case of transient parosmia [8]. Further smell and taste dysfunctions have been signaled in the context of post-commercial surveillance, for instance in the UK, 802 cases of anosmia, parosmia, and hyposmia in over 25 million administered doses of Oxford-AstraZeneca have been reported, compatible with a possible rare adverse event [10]. The persistence of hyposmia in our patient could be explained by the OB volume reduction seen in the MRI, similar to previously reported findings in post-infection olfactory loss [11], even though the advanced age of the patient needs to be taken into account as age related reduction of OB volume is a well-known physiological process and no previous MRI of the patient was available for comparison [5]. On the other hand, the presence of transient tinnitus could also indicate an anxiety reaction to the vaccination [2], as anxiety is a well-known trigger for tinnitus [12]. Due to the wide spread of SARS-CoV-2, which is responsible of COVID-19, we cannot even exclude the possibility that the patient had been infected immediately after vaccination, since the symptoms he reported were similar to the most common COVID-19-related ones [13]. By the way, even if we are aware of the risk of obtaining false-negative results, the swab did not reveal SARS-CoV-2 infection.

The benefits of the anti-COVID-19 vaccination clearly outweigh the risk of rare otolaryngologic adverse events, which are transient in most cases. To the best of our knowledge, no report of simultaneous olfactory and audiovestibular symptoms after vaccination with complete clinical and radiological investigations has been published yet. Further accurate studies are needed to better define the possible auto-immunological mechanisms.
